# Genetic transformation technologies for the common dandelion, *Taraxacum officinale*

**DOI:** 10.1186/s13007-021-00760-3

**Published:** 2021-06-09

**Authors:** Kasia Dinkeloo, Araceli Maria Cantero, Inyup Paik, Alexa Vulgamott, Andrew D Ellington, Alan Lloyd

**Affiliations:** Department of Molecular Biosciences, College of Natural Sciences, The University of Texas at Austin, Austin, TX 78712 USA

**Keywords:** Dandelion, Transformation, Transient expression, Protoplast transformation, Taraxacum officinale

## Abstract

**Background:**

*Taraxacum officinale*, or the common dandelion, is a widespread perennial species recognized worldwide as a common lawn and garden weed. Common dandelion is also cultivated for use in teas, as edible greens, and for use in traditional medicine. It produces latex and is closely related to the Russian dandelion, *T. kok-saghyz*, which is being developed as a rubber crop. Additionally, the vast majority of extant common dandelions reproduce asexually through apomictically derived seeds- an important goal for many major crops in modern agriculture. As such, there is increasing interest in the molecular control of important pathways as well as basic molecular biology and reproduction of common dandelion.

**Results:**

Here we present an improved *Agrobacterium*-based genetic transformation and regeneration protocol, a protocol for generation and transformation of protoplasts using free DNA, and a protocol for leaf *Agrobacterium* infiltration for transient gene expression. These protocols use easily obtainable leaf explants from soil-grown plants and reagents common to most molecular plant laboratories. We show that common markers used in many plant transformation systems function as expected in common dandelion including fluorescent proteins, GUS, and anthocyanin regulation, as well as resistance to kanamycin, Basta, and hygromycin.

**Conclusion:**

Reproducible, stable and transient transformation methods are presented that will allow for needed molecular structure and function studies of genes and proteins in *T. officinale*.

## Introduction

The use of model organisms to understand genome-to-phenome problems has enjoyed undisputed success for decades. The ability to study and understand biological phenomena has been driven by the development of technologies using these models as foils for the rest of life. There has always been work using “non-model” organisms, but often this work has been hampered by the paucity of tools to manipulate genes and genomes, or to even know what genes are present.

## Why study dandelion?

*Taraxacum officinale* or the common dandelion is a weedy perennial that is extremely widely distributed in the biosphere. Although it’s center of distribution is Eurasia, it occurs from the tropics to the temperate zones in the northern and southern hemispheres. While it is native to the old world, like most weeds it has spread with human activity. Dandelion is a minor vegetable crop with the greens eaten cooked or fresh and the roots made into tea. The blossoms are made into the widely familiar Dandelion Wine. Dandelion can be immediately identified by anyone who has gardened or tried to maintain a lawn.

Several labs are working on various aspects of common dandelion biology. Examples include genome size and ploidy determinations for hundreds of accessions [1]; mapping of genes that control aspects of apomixis [2–4]; and determining the bioactivity of extracts and compounds in various medically relevant treatments and their possible benefits for human nutrition [5–10].

**Fig. 1:**
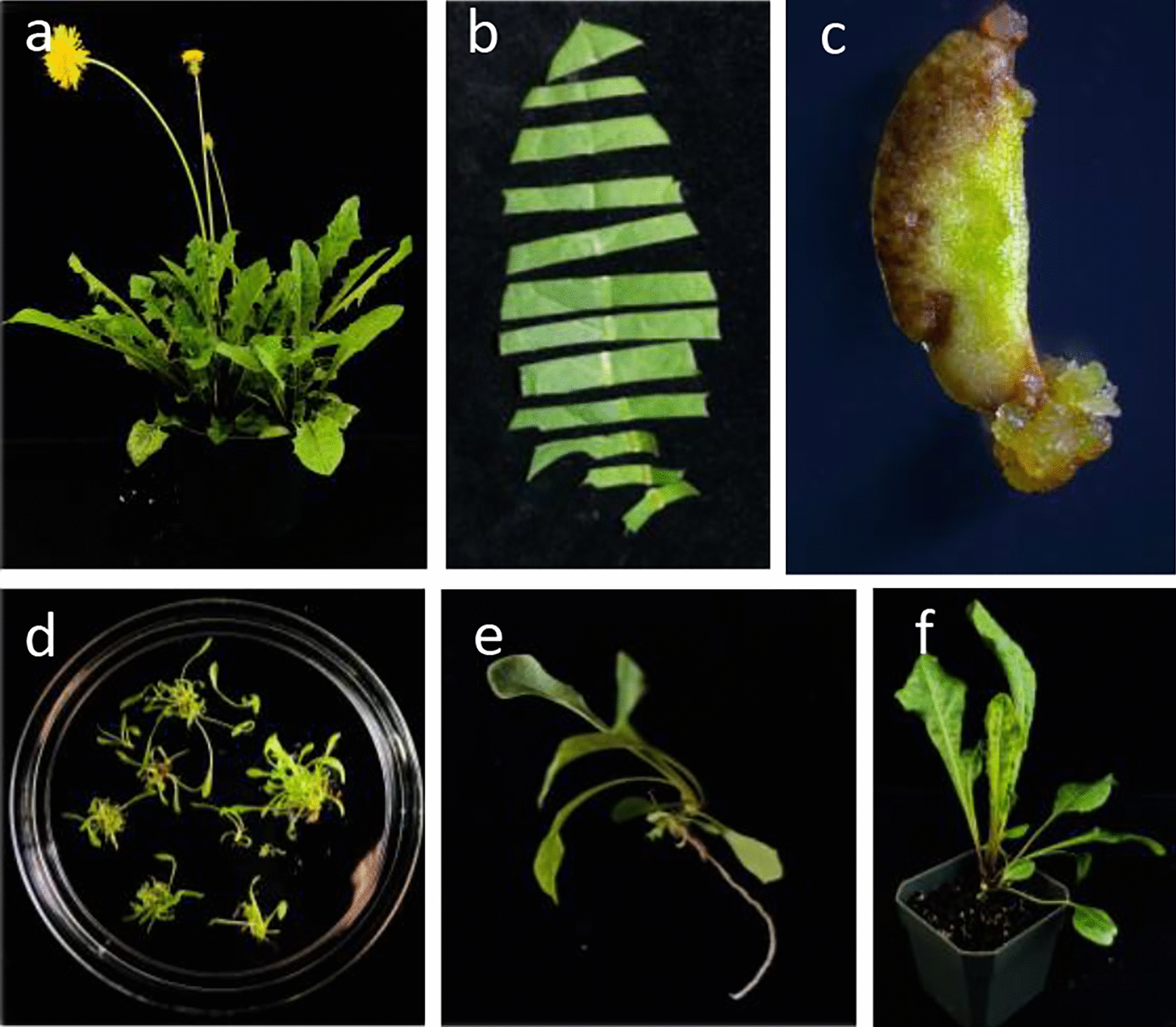
Stages of Dandelion transformation. **a** Flowering dandelion plant grown on soil in a growth chamber. **b** Common dandelion leaf cut into explants for transformation via tissue culture. **c** Transgenic callus growing from a leaf explant. **d** Plantlet stage of transformation in which small groups of leaves grow from the callus. **e** Rooting occurs at variable times during the plantlet stage. **f** Transgenic dandelion are moved to soil after roots are present

Common dandelion is robust. It is easy to culture under growth chamber, greenhouse, and garden conditions (Fig. 1a). Genetic transformation is a must in order to do modern genotype to phenotype studies. There are several published protocols for stable transformation of *Taraxacum kok-saghyz, T. brevicorniculatum, and T. platycarpum* [11–13] and a single publication describes transformation of *T. officinale* [14]. Here, we have modified these protocols for simplification of explant source and in vitro manipulation of common dandelion. We use soil grown leaf piece explants, co-cultivate with *Agrobacterium*, then callus and regenerate shoots on a single hormone regime. We then induce rooting of regenerated shoots on another hormone regime, and subsequently transfer to soil. The process takes as little as 12 weeks from explant to soil and then another six to eight weeks to flower. We have successfully used three available selectable markers: resistance to kanamycin; hygromycin; and Basta. Additionally, Basta herbicide resistance provides for easy and cheap transformant and progeny screening in soil. We have shown that GUS and fluorescent protein markers function well in common dandelion. To provide an additional visual marker, we have shown that the anthocyanin pathway can be upregulated in dandelion with the MYB113 anthocyanin regulator from *Arabidopsis*, resulting in deep purple leaves [15]. To complement stable genetic transformation, we have developed leaf infiltration (à la *Nicotiana benthamiana* [16]) as a method for quickly and transiently assaying gene function. Further, we developed a protocol to perform protoplast transformation using free DNA for possible large-scale screening technologies.

## Materials and methods

### *Agrobacterium* and constructs used in transformation experiments

*Agrobacterium*-based experiments were performed using *Agrobacterium tumefaciens* strain GV3101 pMP90 [17]. The following plasmids were used: pGFPGUSPlus [18]; Addgene plasmid # 64401; contains CaMV35S-driven hygromycin resistance, EGFP reporter, and GUS reporter; pMYB113 [15]; contains NOS promoter driven hygromycin resistance, CaMV35S:Arabidopsis MYB113; pPM-YB [19]; contains plasma membrane localized YFP, Mannopine Synthase Promoter:phosphinothricin (Basta) acetyl transferase; pEZS-CL [20]; a high copy plasmid containing CaMV35S:EGFP.

For the leaf infiltration experiments, we also included the RNA silencing suppressor, P19 in *Agrobacterium* strain GV 3101 [21]. *Agrobacterium* and *E. coli* strains were maintained on LB media with appropriate antibiotics to select for the plasmids and Rifampicin 10 mg/L and Gentamycin 30 mg/L to select for *Agrobacterium* GV3101 pMP90 [17].

### *Agrobacterium*mediated transformation and regeneration of common dandelion

Young leaves (10 to 15 cm long) were excised from soil grown plants and placed in 10% bleach with 0.04% Tween 20 for 10 min for surface sterilization, and then rinsed 4 times in sterile water. The *Agrobacterium* solution was prepared from overnight *Agrobacterium* cultures grown in 5mL LB media under antibiotic selection. These cultures were pelleted via centrifugation at 3000 × g for 10 min. The pellet was resuspended in induction solution (1XMS, 3% Sucrose, 1% Glucose, 0.2 mM acetosyringone, pH 5.2) as a first wash, and then pelleted and resuspended a second time in 5 mL induction solution. The *Agrobacterium* in induction solution was then incubated at 30 °C with agitation for 1 hour. Leaves were cut into strips approximately 0.75 cm long spanning the width of the leaf. The leaf strips were placed into an empty petri dish, and the *Agrobacterium* induction solution was added to the leaf strips with gentle agitation for an incubation period of 5 min. These strips were gently tapped on an empty petri dish to remove excess *Agrobacterium* induction solution, and placed adaxial side up on petri plates containing cocultivation media (1XMS, 3% sucrose, 1% glucose, 0.1 mM acetosyringone + 2 mg/L BAP, 0.1 mg/L NAA, 0.9% agar pH 5.2). Petri dishes were sealed with micropore tape and leaf pieces and *Agrobacterium* were co-cultivated for 2 days in darkness at 26 °C. After co-cultivation, leaf pieces were moved to media containing 1XMS, 3% sucrose, + 2 mg/L BAP, 0.1 mg/L NAA, 300 mg/L Timentin, 0.9% agar pH 5.2 and sealed with parafilm.

After 7 days, the leaf pieces were transferred to shoot induction media (1XMS, 3% sucrose, 2 mg/L BAP, 0.1 mg/L NAA, 0.9% agar, 300 mg/L Timentin, pH 5.7) containing the appropriate antibiotic or herbicide to select for transformed dandelion cells. We used 100 mg/L kanamycin, 50 mg/L hygromycin, or 3 mg/L Basta (phosphinothricin), all from Sigma. When leaves on shoots were approximately 1.5–2 cm long, they were transferred to the same media but with 0.1 mg/L NAA to elicit root differentiation. Shoots with well-formed roots at least 1.5 cm long were transferred to soil (Promix BX; Hummert International) and placed in flats with transparent lids to keep the humidity high. Once new leaf growth was observed, the lids were gradually removed.

### Genotyping of transgenic dandelion; inheritance of TDNA in progeny of primary transformants

PCR amplification of sequences on the *Agrobacterium* TDNA was used to assess whether potential regenerated transformants indeed contained the transgene. PCR primers and the size of the expected DNA fragments are listed in Table 1. Genomic DNA was isolated from dandelion using the CTAB method [22], using 10–15 mg of tissue from leaves that emerged after the plants were transferred to soil or from tissue from the next generation seedlings. To test for markers in transgenic progeny, seeds were sown in 10 cm square pots on the soil surface (promix BX) and grown at 24 °C under fluorescent lights with a 16/8 h light/dark cycle.

**Table 1:** List of primers for genotyping analysis of dandelion

Primer name	The sequence (5′-3′)	Length of product (bp)
ACTIN-F	CGTCGATCTCAAGGATGTTGTC	120
ACTIN-R	GGAGCTTTGAGAAGAACCAACG	
YFP-F	ATGGTGAGCAAGGGCG	300
YFP-R	TTGTACAGCTCGTCCATGC	
BASTA-F	AAACCCACGTCATGCCAGTT	343
BASTA-R	AAGCACGGTCAACTTCCGTA	

### Basta herbicide resistance assay

For Basta herbicide resistance assays, seeds were sown as above and 10 to 14 day old wildtype or Basta resistant seedlings grown in 2.5 inch pots were treated with 25 ml of 3 mg/l Basta, results were collected 7 days after exposure.

### *Agrobacterium* mediated leaf-infiltration for transient gene expression

The bacteria grown as above and were harvested by centrifugation for 3 min at 3000 × g. The pellets were rinsed by resuspension in the same volume of infiltration buffer (10 mM MgCl_2,_ 10 µM acetosyringone) and centrifuged again for 3 min at 3000 × g. Pellets were again resuspended in infiltration buffer. The OD_600_ was measured and each strain was diluted to OD_600_ of 0.1 with infiltration buffer. Each infiltration experiment contained two *Agrobacterium* strains: one strain contained the transient gene expression construct and the other contained the RNA silencing suppressor, P19 [21].

Young dandelion plants approximately four weeks of age were used for infiltration. The bacterial suspension was infiltrated into the abaxial leaf surface using a 1 ml tuberculin syringe without needle in the method of Vaghchhipawala et al. [16]. Briefly, the syringe tip is held tightly to the abaxial leaf surface and a gloved finger is held on the opposite adaxial side while the syringe plunger is gently but firmly pushed forcing the *Agrobacterium* solution into the leaf interior. 3–5 days post infiltration the leaf was excised from the plant and transient gene expression was tested by either GUS staining according to Jefferson et al. [23], observing YFP fluorescence under a fluorescent microscope, or visual inspection for anthocyanin accumulation in the infiltrated area.

### Protoplast preparation

Two incubation regimes were tested: 30 °C for 3 hours and 22 °C for 15 to 17 h. For the 30 °C treatment, the enzyme solution was prepared as follows, 20 mM MES (pH 5.7), 0.5 M mannitol, 20 mM KCl, cellulase R10 1.0% wt/vol (Yakult Pharmaceutical Industry Co., Ltd), macerozyme R10 0.5% wt/vol (Yakult Pharmaceutical Industry Co., Ltd). This enzyme solution was heated to 60 °C for 5 minutes and cooled to room temperature. CaCl_2_ was added to 10 mM and BSA to 0.1%. The 22 °C treatment used the same solutions except for 0.45% wt/vol cellulase R10, 0.2% wt/vol macerozyme R10.

Protoplasts were prepared from the 3rd to 5th leaves (approximately 5 to 6 cm long) from 3 to 4-week old dandelion plants. Dandelion leaves were detached from the plants and lightly scratched with sandpaper (3M 413Q, 220 grit) on the abaxial side before immediate submersion in 10 ml of enzyme solution. They were incubated at either 22 °C overnight or 30 °C for 3 h in the dark in 10 cm petri dishes. After the incubation period, 10 ml of W5 solution (2 mM MES pH 5.7, 154 mM NaCl, 125 mM CaCl_2_, 5 mM KCl) was then added to the enzyme solution to stop the reaction. The 20 ml reaction mix was filtered through a 100 µm cell strainer (Fisher Scientific., Cat# 22-363-549) and split into two 15 ml round bottom tubes. The protoplasts were collected by centrifuging for 2 min at 1000 × g. The supernatant was removed by pipetting as much liquid as possible while leaving the pellet intact in the tube. The green pellets were resuspended by adding 2 ml of W5 solution and the tubes were incubated on ice for 30 min.

The cell number was counted using a hemocytometer. The protoplasts were resuspended at 5 × 10^5^ cells/ml in MMG solution (4 mM MES pH 5.7, 0.4 M mannitol, 15 mM MgCl_2_) prior to transformation. Protoplast viability after isolation was analyzed using the fluorescein diacetate staining method described by Larkin [24].

### Free DNA delivery to protoplasts

A total of 10 µg of plasmid DNA (pEZS-CL) at 1.5 to 3 µg/µL concentration was added to 100 µl of MMG resuspended protoplasts and this was gently mixed before adding 100 µl of PEG transformation solution (30% wt/vol PEG4000 (Sigma Aldrich., Cat# 95904), 0.2 M mannitol, 100 mM CaCl2) for 5 min. The transformation reaction was stopped by adding 20 ml of W5 solution and the protoplasts were collected by centrifugation for 2 min at 1000 g. Transformed protoplasts were incubated at 22 °C for 15 to 17 h in WI solution (4 mM MES pH 5.7, 0.5 M mannitol, 20 mM KCl) before the GFP signal was detected.

### Observation of fluorescent protein or GUS expression

YFP or GFP protein fluorescence was observed on an Olympus BX53 microscope with a YFP or GFP filter at 10x with a 1 second exposure. GUS staining was done according to Jefferson et al. [23].

## Results

### Stable transformation and regeneration

**Fig. 2:**
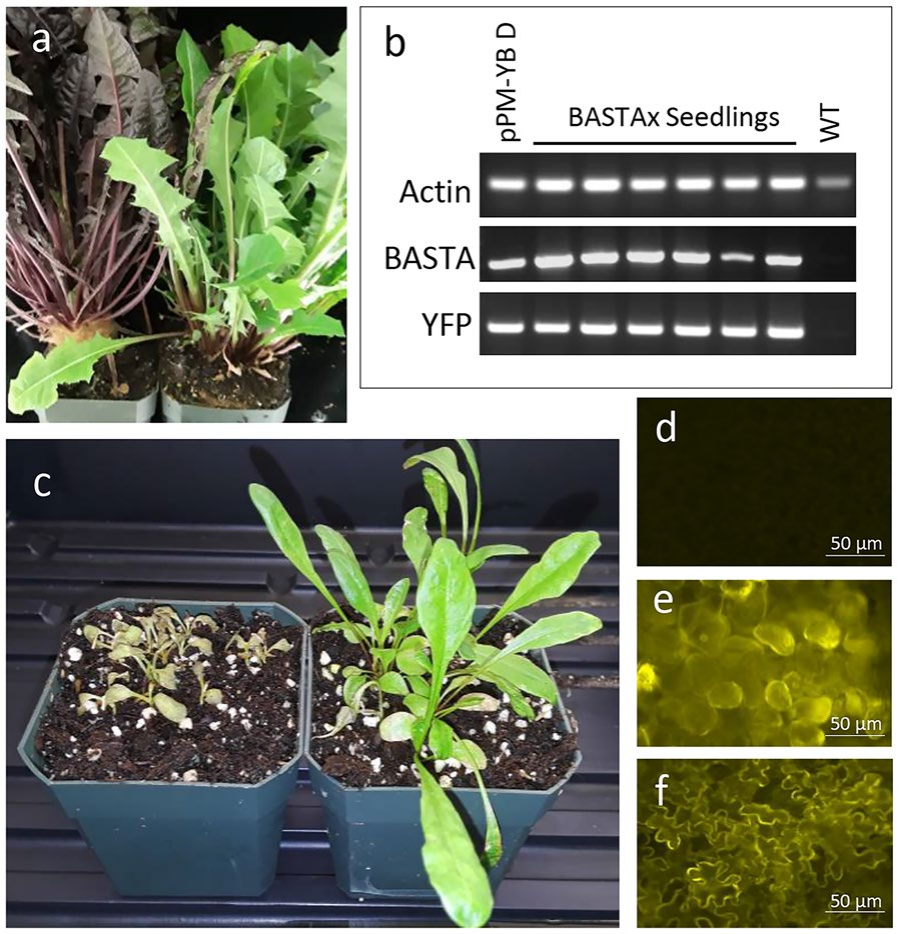
Inheritance of TDNA in progeny of primary transformants. **a** Induction of red/purple anthocyanin pigment from expression of the *Arabidopsis* MYB113 transcription factor shown in transgenic dandelion (left) versus no pigment in wild type plants (right). **b** Amplification of *Agrobacterium* TDNA sequences for Basta resistance and YFP present in transgenic parental plant and seedlings and absent in WT. **c** WT seedlings (left) are killed by treatment with Basta herbicide, Basta-resistant seedlings (right) are unaffected. **d** Fluorescent microscopy of WT shows no fluorescence. **e**, **f** Transgenic seedlings show YFP fluorescence in mesophyll and epidermal cells respectively

In order for any plant to truly submit to molecular analysis there must be a reliable transformation and regeneration protocol to produce stable whole transgenic plants. We developed a protocol modified from previous *Taraxacum* researchers [11–14]. We began by trying to use aseptic explants from seedlings germinated and grown in sterile culture. While we were able to transform explants from these seedlings, it is much less labor and resource intensive to surface sterilize leaves from soil grown plants (Fig. 1a). Explants were prepared but cutting sterilized leaves into strips (Fig. 1b). Uninoculated explants will differentiate shoots in as little as 20 days in the absence of selection on MS media containing 2 mg/L BAP, 0.1 mg/L NAA. In the presence of Basta and hygromycin selection, most uninoculated explants do not give rise to callus or differentiate. Under kanamycin selection some explants will give rise to bleached white callus and leaves. With *Agrobacterium* inoculation and selection, we found that transgenic callus most often emerged from the explant edges at the site of major veins after 15 to 20 days (Fig 1c). Shoots typically emerged from these calli in 5 to 6 weeks (Fig. 1d, e). Three selection markers were successfully used to select for transgenic plant cells and plants: kanamycin, hygromycin, and Basta resistance. The percentage of leaf explants that produced transgenic callus and shoots was: ~ 32% for Basta, ~ 23% for kanamycin, and ~ 30% for hygromycin (Table 2). Most explants gave multiple independent foci of transformation. As a rule, we only retained one transgenic plant from each explant to ensure we were observing independent events. Transgenic shoots were rooted on MS media with 0.1 mg/L NAA before being transferred to soil. The timing for rooting of shoots was quite variable, from as short as 6 weeks to as long as 16 weeks. Genotyping was done on leaves that were newly emerged after the transition to soil, although transformation of dandelion using the *Arabidopsis* MYB113 transcription factor was readily visible with the naked eye by observing the induction of red/purple anthocyanin pigment synthesis (Fig. 2a). We have repeated these transformation/regeneration experiments many times, however, transformation frequency data was only collected from 2 experiments with each for Basta and kanamycin selection, and a single experiment for hygromycin selection (Table 2).

**Table 2:** Transformation efficiency of leaf explants under different selection

Selection	Explant #	Transformant #	T efficiency%
Basta I	94	30	32
Basta II	36	11	31
Kanamycin I	113	21	19
Kanamycin II	36	10	28
Hygromycin	89	27	30
		Average % ± st. dev.	27.9 ± 7.23

The seeds of transgenic dandelions were collected and germinated to understand the heritability of transgenes. Fig. 2b presents the inheritance of the Basta resistance marker gene and YFP marker gene in transgenic offspring. Amplified bands were separated and observed using agarose gel electrophoresis, and bands confirming the presence of YFP and Basta were apparent for the parental plant and all offspring, but not for the wild type plant. To confirm the activity of the transgenes, wildtype seedlings and Basta resistant seedlings were treated with 3 mg/l Basta. Wild-type seedlings are killed by the herbicide, while transgenic progeny are resistant (Fig. 2c). The seedlings were also tested for YFP expression where fluorescent microscopy shows the transgenic seedlings expressing active YFP fluorescence, and the wild type seedlings do not (Fig. 2d, e, f).

### *Agrobacterium* mediated leaf-infiltration for transient gene expression

**Fig. 3:**
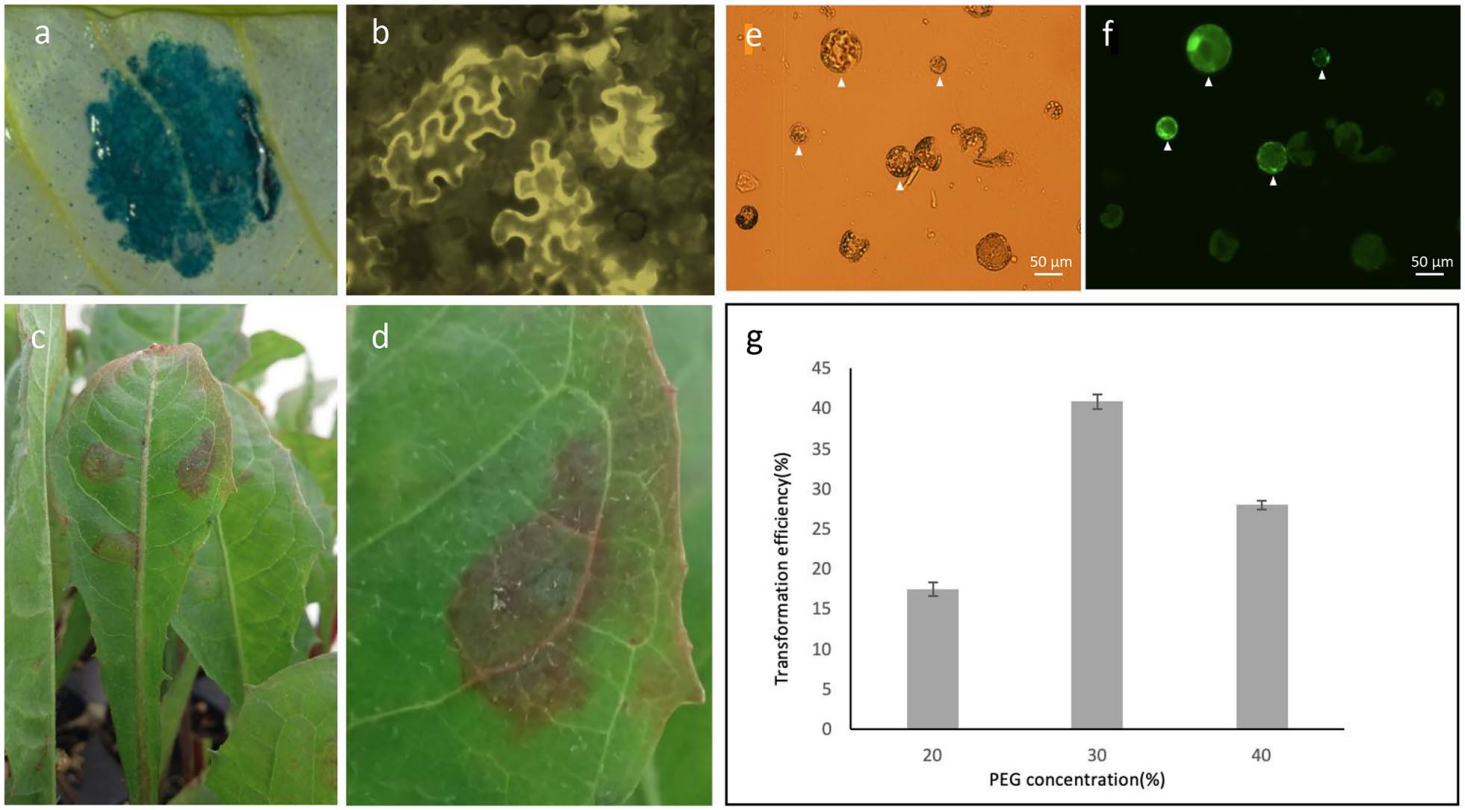
Transient gene expression and protoplast transformation in Common Dandelion. When using different reporter constructs in *Agrobacterium*, transient expression is achieved as shown by: **a** GUS staining, **b** YFP fluorescence, and **c** anthocyanin accumulation. **e** Transformed dandelion protoplasts shown with light microscopy. **f** fluorescent microscopy shows activity of the YFP reporter gene in transgenic protoplasts. **g** Transformation efficiencies resulting from different concentrations of PEG. Each PEG concentration experiment was repeated three times. Error bars represent standard deviation

Infiltration of leaves with *Agrobacterium tumefaciens* is often used as a transient assay for gene expression studies. While *Nicotiana benthamiana* is the most commonly used species for this technology, methods have been worked out for many others [25, 26]. The protocol we present here is based on *N. benthamiana* methods [16]. We successfully infiltrated constructs into the abaxial side of dandelion leaves. We were able to observe both YFP and GUS reporter gene expression (Fig. 3) 3 to 5 days after treatment.

### Protoplast transformation

Protoplast transformation can provide a means to test transformation constructs or to screen inducers or inhibitors, or perform other assays in a fast and high throughput manner [9, 27]. To establish a protoplast transformation system, we examined whether common dandelion leaf tissue could be efficiently digested with the enzymes commonly used for mesophyll protoplast isolation in *Arabidopsis* [9]. With slight modifications in enzyme concentrations and buffer conditions, we were able to isolate intact protoplasts from 3 to 4-week old dandelion leaves (Fig. 3e). As reported previously for switchgrass [27] the age of the plants affected the cell wall digestion efficiency, with leaves from older plants being more difficult to digest and producing a lower yield of protoplasts. Therefore, we only selected the 3rd to 5th leaf from 3 to 4-week old dandelions for the preparation of protoplasts. The protoplasts were assayed for viability using fluorescein diacetate staining, with an average of 75.2% (±1.7) of protoplasts maintaining viability after extraction (Table 3). We then performed PEG-based protoplast transformation as described above and observed the cells for fluorescence at 15 to 17 h post transformation (Fig. 3f). We examined the effect of three different concentrations of PEG (20, 30, and 40%) on protoplast transformation efficiency because others have reported that PEG concentration was an important variable [27]*.* We found that protoplasts were successfully transformed with all concentrations. The 30% PEG protocol gave up to 40% transformation efficiency, while 20% and 40% PEG protocols provided a transformation efficiency of up to 25% (Fig. 3g). These efficiencies are sufficient for most assay applications and as they stand can easily be adapted for automated liquid handling.

**Table 3:** Protoplast viability

Replicate	Viable Protoplasts	Total Protoplasts	Viability (%)
1	66	86	76.7
2	58	77	75.3
3	69	94	73.4
		Average %	75.2
		±	1.7

## Discussion

There are many important qualities that make the common dandelion an appealing research subject. It is easy to cultivate in many environmental conditions from growth chambers and greenhouses to the field, and it produces many progeny per composite flower head. Other qualities include: a largely apomictic lifestyle, its use as a vegetable for greens, the use of its roots in teas and flowers in wine, its use in traditional and modern medical studies, and finally, the ability to make latex and its close relationship to the latex/rubber producing Russian dandelion.

We note here that increasing numbers of “non-model” plants are being tamed for specific genomic engineering opportunities often based on desirable idiosyncratic properties. These include developmental patterns, physiologies, or secondary metabolic pathways. The common dandelion abounds in all of these with its rosette form with taproot, its family-typical composite flower head, its largely apomictic reproduction mode, and its many potentially valuable secondary metabolic pathways.

As an example, several groups are working on the closely related Russian or rubber dandelion, *Taraxacum kok-saghyz* (a few recent examples include: [28–33]**,** with the goal to develop it as a natural rubber production crop to backup or replace the rubber tree, a crop with a troubled past and future [34, 35]. Rubber production from latex in Russian dandelion is often extolled in popular media [36], however, *T. kok-saghyz* is a species with a very narrow distribution, endemic only in southeast Kazakhstan, and it has exacting culture requirements [34]. As a latex-producer related to Russian dandelion, the “genome-enabling” of common dandelion could lead to synthetic biology approaches where efficient rubber production is engineered into this much more agriculturally facile species, or its facile properties engineered or hybridized into the Russian dandelion. The natural rubber market produced 14 million metric tons in 2018 up from 8.3 million metric tons in 2004 and the demand is expected to continue to rise [37, 38]. Much could be gained by leveraging common dandelion in the cause of an alternative commercial source for natural rubber.

In order to genomically enable the common dandelion, many tools will need to be developed for this species. These tools would include robust annotated transcriptome and genome sequences, the ability to do traditional genetics, the ability to facilely transform the plant both stably and transiently, and the ability to create knock-down or loss-of-function mutants.

Here we present simple methods for transient transformation of dandelion leaves and protoplasts, and for stable transformation and regeneration of plants. We have shown that the common dandelion is amenable to common techniques used in many other species. A few improvements our protocol offers include a dandelion explant source that is easily grown on soil in conditions we use for *Arabidopsis thaliana*, and one hormone regime for stable transformation and regeneration and another for rooting shoots. Our data show that at least three selection regimes are possible including kanamycin, hygromycin, and Basta resistance. We also found that standard markers function as expected in dandelion including: YFP, GFP, GUS, and MYB regulation of the anthocyanin pathway. In this manuscript we present transformation efficiency, timeline, and optimization efforts, providing important information for those attempting transformation of common dandelion

In conclusion, we describe a set of protocols that will help make the common dandelion amenable to modern techniques used in other model species. Any lab that has the facilities to do molecular work in *Arabidopsis* or *Nicotiana*, for example, will be able to perform the same work in common dandelion.

## Data Availability

The datasets used and/or analyzed during the current study are available from the corresponding author on reasonable request.
